# Supplementary material for: Multiple sclerosis risk variants regulate gene expression in innate and adaptive immune cells
Source: Life Sci Alliance. 2020 Jun 9;3(7):e202000650. doi: 10.26508/lsa.202000650 (PMC7283543; doi:10.26508/lsa.202000650)
Supplement: Supplementary file 1 [file LSA-2020-00650_Supplemental_Data_1.pdf]

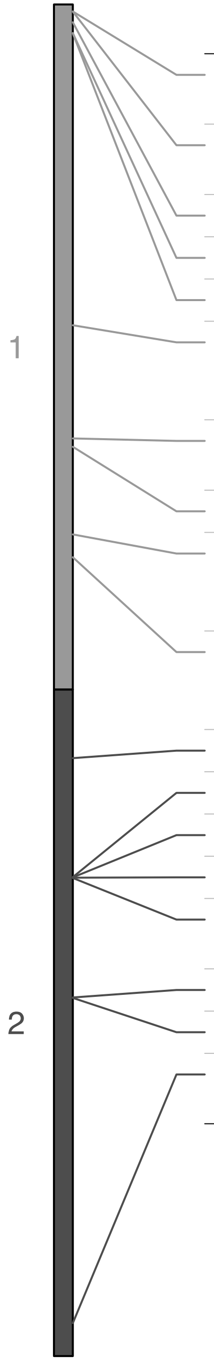

Regulated gene	2011 SNP	2011M SNP	2013 SNP	Monocyte eQTL	NK cell eQTL	B cell eQTL	CD4 cell eQTL	CD8 cell eQTL
TNFRSF14	rs4648356	–	rs3748817	N	rs4648356<br>rs3748817	N	rs4648356<br>rs3748817	rs3748817
FAM213B	rs4648356	–	rs3748817	rs4648356<br>rs3748817	N	rs3748817	N	N
TNFRSF25	–	–	rs3007421	N	N	N	N	rs3007421
PGD	–	rs10492972	–	rs10492972	N	N	rs10492972	rs10492972
PEX14	–	rs10492972	–	rs10492972	N	N	N	N
CD58	rs1335532	rs2300747	rs6677309	N	N	rs1335532<br>rs2300747<br>rs6677309	N	N
FCRL3	rs3761959	–	rs2050568	N	rs3761959<br>rs2050568	rs3761959<br>rs2050568	rs3761959<br>rs2050568	rs3761959<br>rs2050568
SLAMF7	–	–	rs35967351	N	N	rs35967351	N	N
RGS1	rs1323292	rs2760524	rs1359062	rs1323292<br>rs2760524<br>rs1359062	N	rs1323292<br>rs2760524<br>rs1359062	N	N
GPR25	rs7522462	rs12122721	rs55838263	N	N	N	N	rs7522462<br>rs12122721<br>rs55838263
ADCY3	–	–	rs4665719	rs4665719	N	N	rs4665719	N
PNO1	–	–	rs7595717	rs7595717	N	N	N	N
PPP3R1	rs7595037	–	–	N	N	rs7595037	N	N
PLEK	–	–	rs7595717	N	rs7595717	N	N	rs7595717
APLF	rs7595037	rs7592330	–	N	N	N	rs7595037<br>rs7592330	N
MERTK	–	–	rs17174870	rs17174870	N	N	rs17174870	N
TMEM87B	–	–	rs17174870	N	rs17174870	N	N	N
SP140	rs10201872	–	rs9989735	rs10201872<br>rs9989735	N	N	N	rs9989735

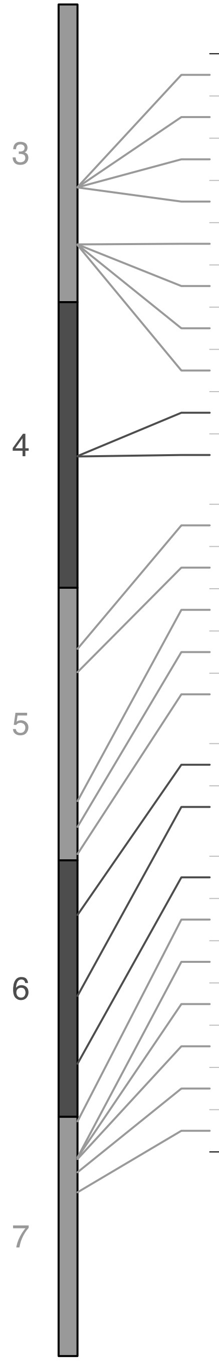

Regulated gene	2011 SNP	2011M SNP	2013 SNP	Monocyte eQTL	NK cell eQTL	B cell eQTL	CD4 cell eQTL	CD8 cell eQTL
RNU4–62P	–	–	rs1920296	N	N	rs1920296	N	rs1920296
IQCB1	–	–	rs1920296	rs1920296	rs1920296	rs1920296	rs1920296	rs1920296
EAF2	–	–	rs1920296	N	N	rs1920296	N	N
SLC15A2	–	–	rs1920296	N	N	rs1920296	N	N
RNU2–31P	–	rs4680534	–	N	N	N	rs4680534	N
SCHIP1	–	–	rs1014486	rs1014486	N	N	N	N
IL12A	rs2243123	–	–	N	N	rs2243123	N	N
C3orf80	rs2243123	–	–	N	N	N	N	rs2243123
NFKB1	–	–	rs7665090	N	N	N	N	rs7665090
MANBA	rs228614	–	rs7665090	rs228614	N	rs7665090	rs7665090	rs228614<br>rs7665090
PTGER4	–	–	rs6880778	rs6880778	rs6880778	N	N	N
ANKRD55	–	–	rs71624119	N	N	N	rs71624119	N
NDFIP1	–	–	rs35952555	N	rs35952555	rs35952555	N	rs35952555
UBLCP1	rs2546890	–	rs2546890	N	N	N	rs2546890	N
RGS14	rs4075958	–	rs4976646	rs4075958<br>rs4976646	N	N	N	N
ETV7	–	–	rs941816	N	rs941816	N	rs941816	rs941816
BACH2	rs12212193	–	rs72928038	N	N	N	rs12212193<br>rs72928038	rs72928038
AHI1	rs11154801	–	rs11154801	rs11154801	rs11154801	rs11154801	rs11154801	rs11154801
CARD11	–	–	rs1843938	N	N	N	N	rs1843938
HOXA–AS2	–	–	rs706015	rs706015	N	N	N	rs706015
HOXA11	–	–	rs706015	N	N	N	rs706015	N
JAZF1	–	–	rs917116	rs917116	N	N	rs917116	N
ELMO1	–	–	rs60600003	N	N	N	N	rs60600003
IKZF1	–	–	rs201847125	N	rs201847125	N	N	N

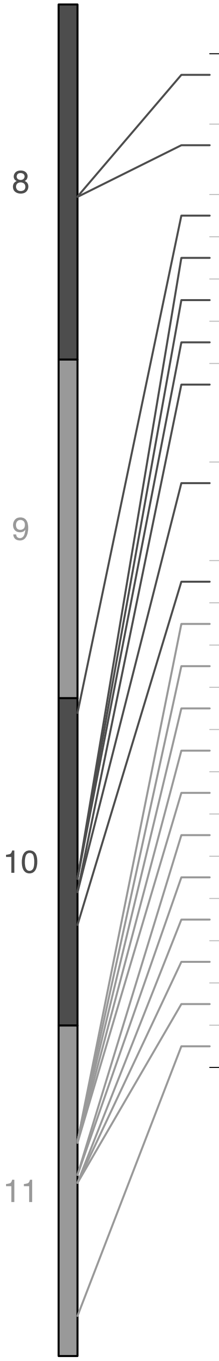

Regulated gene	2011 SNP	2011M SNP	2013 SNP	Monocyte eQTL	NK cell eQTL	B cell eQTL	CD4 cell eQTL	CD8 cell eQTL
PKIA	rs1520333	–	rs1021156	N	N	rs1021156	rs1520333<br>rs1021156	rs1021156
ZC2HC1A	rs1520333	–	rs1021156	rs1520333<br>rs1021156	rs1021156	rs1021156	rs1021156	rs1021156
IL2RA	–	–	rs2104286	N	N	N	N	rs2104286
FUT11	–	–	rs2688608	N	N	N	rs2688608	N
NDST2	–	–	rs2688608	N	N	N	rs2688608	rs2688608
CAMK2G	–	–	rs2688608	rs2688608	N	rs2688608	rs2688608	rs2688608
ZMIZ1	rs1250550	rs1250542	rs1782645	N	rs1250550<br>rs1250542<br>rs1782645	N	N	rs1250550<br>rs1250542
PPIF	rs1250550	rs1250542	rs1782645	N	N	N	N	rs1250550<br>rs1250542<br>rs1782645
HHEX	rs7923837	–	rs7923837	N	rs7923837	N	N	N
DDB2	–	–	rs7120737	rs7120737	N	N	N	N
ACP2	–	–	rs7120737	rs7120737	rs7120737	rs7120737	N	rs7120737
NR1H3	–	–	rs7120737	N	rs7120737	N	N	N
AGBL2	–	–	rs7120737	N	N	N	N	rs7120737
PTPRJ	–	–	rs7120737	rs7120737	N	N	rs7120737	N
CD6	–	–	rs34383631	N	N	N	rs34383631	N
CD5	–	–	rs34383631	N	N	N	rs34383631	N
FKBP2	–	–	rs694739	rs694739	N	N	rs694739	rs694739
GPR137	–	–	rs694739	N	rs694739	N	N	N
CCDC88B	–	–	rs694739	rs694739	N	N	N	N
RNU6–376P	–	–	rs533646	rs533646	N	N	N	N

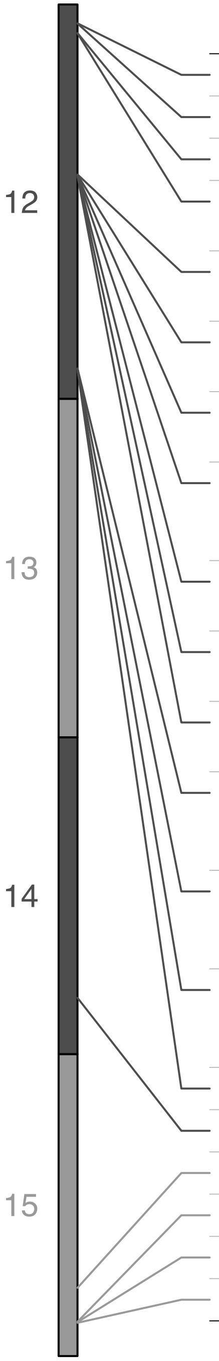

Regulated gene	2011 SNP	2011M SNP	2013 SNP	Monocyte eQTL	NK cell eQTL	B cell eQTL	CD4 cell eQTL	CD8 cell eQTL
LTBR	–	–	rs12296430	N	N	N	rs12296430	N
TAPBPL	rs1800693	–	rs1800693	N	N	N	rs1800693	N
CLEC2D	–	–	rs11052877	N	N	rs11052877	N	N
CLECL1	rs10466829	–	rs11052877	rs10466829<br>rs11052877	rs10466829	N	rs10466829<br>rs11052877	rs10466829<br>rs11052877
INHBC	–	rs703842	rs201202118	N	N	N	rs703842<br>rs201202118	N
PIP4K2C	–	rs703842	rs201202118	N	N	rs703842<br>rs201202118	N	N
TSPAN31	–	rs703842	rs201202118	N	N	N	rs703842<br>rs201202118	rs703842<br>rs201202118
METTL21B	rs12368653	rs703842	rs201202118	rs12368653<br>rs703842<br>rs201202118	rs12368653<br>rs703842<br>rs201202118	rs703842<br>rs201202118	rs12368653<br>rs703842<br>rs201202118	rs12368653<br>rs703842<br>rs201202118
TSFM	–	rs703842	rs201202118	rs703842<br>rs201202118	N	N	N	N
AVIL	–	rs703842	rs201202118	N	N	N	N	rs703842<br>rs201202118
XRCC6BP1	rs12368653	–	rs201202118	N	N	rs12368653	N	rs12368653<br>rs201202118
ABCB9	rs949143	rs1790100	rs7132277	N	N	N	rs949143<br>rs1790100<br>rs7132277	rs949143<br>rs1790100<br>rs7132277
ARL6IP4	rs949143	rs1790100	rs7132277	N	rs949143<br>rs7132277	N	N	rs949143<br>rs1790100<br>rs7132277
CDK2AP1	rs949143	rs1790100	rs7132277	rs1790100<br>rs7132277	rs949143	rs1790100	rs1790100<br>rs7132277	rs949143<br>rs1790100<br>rs7132277
SBNO1	rs949143	–	–	N	rs949143	N	N	N
GPR65	rs2119704	–	–	N	rs2119704	N	N	N
CTSH	–	–	rs59772922	rs59772922	N	N	N	N
IQGAP1	–	–	rs8042861	rs8042861	rs8042861	N	rs8042861	rs8042861
CRTC3	–	–	rs8042861	N	N	rs8042861	N	N
BLM	–	–	rs8042861	rs8042861	N	N	N	N

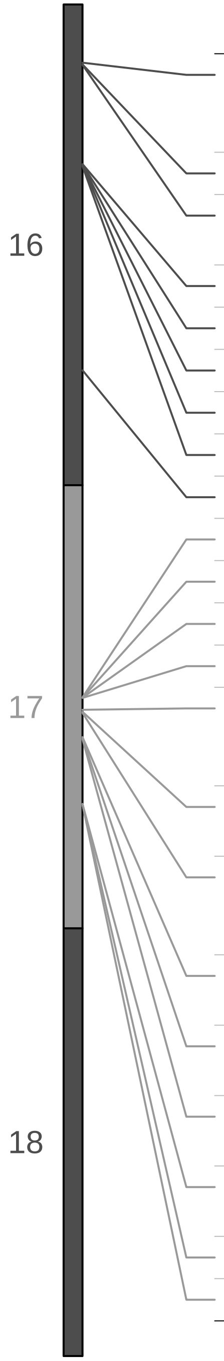

Regulated gene	2011 SNP	2011M SNP	2013 SNP	Monocyte eQTL	NK cell eQTL	B cell eQTL	CD4 cell eQTL	CD8 cell eQTL
DEXI	rs7200786	rs12708716	rs12927355	rs7200786<br>rs12708716<br>rs12927355	N	N	N	rs12927355
TNP2	–	rs7191700	–	N	rs7191700	N	N	N
RMI2	–	rs7191700	rs6498184	N	N	rs7191700<br>rs6498184	rs7191700<br>rs6498184	rs7191700<br>rs6498184
INO80E	–	–	rs7204270	N	N	rs7204270	N	N
TBX6	–	–	rs7204270	N	N	N	N	rs7204270
GDPD3	–	–	rs7204270	N	N	N	N	rs7204270
MAPK3	–	–	rs7204270	N	N	rs7204270	rs7204270	N
TBC1D10B	–	–	rs7204270	N	rs7204270	N	N	N
CDH1	–	–	rs1886700	N	N	N	rs1886700	N
ZPBP2	–	–	rs12946510	N	N	rs12946510	N	N
GSDMB	–	–	rs12946510	N	rs12946510	rs12946510	rs12946510	rs12946510
ORMDL3	–	–	rs12946510	N	rs12946510	rs12946510	rs12946510	rs12946510
GSDMA	–	–	rs12946510	N	N	N	rs12946510	N
GHDC	rs9891119	rs744166	rs4796791	rs9891119<br>rs744166<br>rs4796791	N	N	N	N
PSMC3IP	rs9891119	–	rs4796791	N	rs9891119<br>rs4796791	N	N	N
RAMP2	rs9891119	rs744166	rs4796791	N	N	N	N	rs9891119<br>rs744166<br>rs4796791
EFCAB13	–	rs4239162*	rs4794058	rs4239162<br>rs4794058	N	rs4794058	N	N
NPEPPS	–	rs4239162*	rs4794058	rs4239162<br>rs4794058	N	N	N	N
TBKBP1	–	rs4239162*	rs4794058	rs4239162<br>rs4794058	rs4239162<br>rs4794058	rs4239162<br>rs4794058	rs4239162<br>rs4794058	rs4239162<br>rs4794058
TUBD1	rs180515	–	rs8070345	N	rs180515<br>rs8070345	rs180515	rs180515	N
RPS6KB1	rs180515	–	–	N	N	rs180515	N	N
RNFT1	rs180515	–	rs8070345	N	N	rs180515	N	rs8070345

*Included as a proxy (r^2 = 0.81) for SNP rs8070463 from the 2011M study

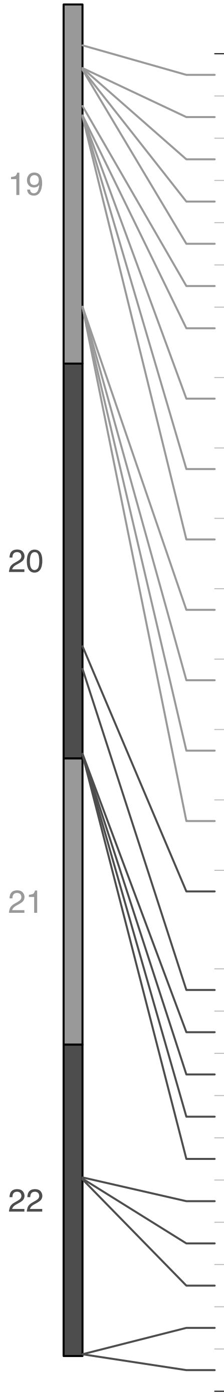

Regulated gene	2011 SNP	2011M SNP	2013 SNP	Monocyte eQTL	NK cell eQTL	B cell eQTL	CD4 cell eQTL	CD8 cell eQTL
TNFSF14	rs1077667	–	rs1077667	rs1077667	N	N	N	N
TYK2	rs8112449	–	–	N	N	N	N	rs8112449
CDC37	rs8112449	–	–	rs8112449	N	N	N	N
SLC44A2	–	–	rs2288904	N	rs2288904	N	rs2288904	rs2288904
ILF3	rs8112449	–	–	N	N	N	N	rs8112449
SLC35E1	–	–	rs1870071	N	N	N	N	rs1870071
MAST3	rs874628	–	rs11554159	rs874628<br>rs11554159	N	N	N	N
IFI30	rs874628	–	rs11554159	rs874628<br>rs11554159	N	N	rs874628<br>rs11554159	N
MPV17L2	rs874628	–	rs11554159	N	N	rs874628<br>rs11554159	N	rs874628<br>rs11554159
KIAA1683	rs874628	–	rs11554159	N	rs874628<br>rs11554159	N	N	N
SLC6A16	rs2303759	–	rs8107548	rs2303759<br>rs8107548	N	N	N	N
CD37	rs2303759	–	rs8107548	N	rs2303759<br>rs8107548	N	rs2303759<br>rs8107548	rs2303759<br>rs8107548
TEAD2	rs2303759	–	rs8107548	N	N	rs2303759<br>rs8107548	N	N
DKKL1	rs2303759	–	rs8107548	N	N	rs2303759<br>rs8107548	N	N
CD40	rs2425752	rs6074022	rs4810485	rs2425752<br>rs6074022<br>rs4810485	N	rs2425752	N	N
SLC9A8	–	–	rs17785991	rs17785991	N	N	N	N
STMN3	–	–	rs2256814	N	N	rs2256814	rs2256814	rs2256814
LIME1	–	–	rs2256814	N	N	N	rs2256814	rs2256814
SLC2A4RG	–	–	rs2256814	N	N	N	N	rs2256814
ZBTB46	rs6062314	–	rs6062314	N	N	rs6062314	N	N
PPIL2	rs2283792	–	rs2283792	N	N	N	rs2283792	N
MAPK1	rs2283792	–	rs2283792	N	N	N	N	rs2283792
TOP3B	rs2283792	–	rs2283792	N	N	rs2283792	rs2283792	N
SCO2	–	–	rs470119	N	N	N	rs470119	N
TYMP	–	–	rs470119	rs470119	N	N	rs470119	N
