# Supplementary figures and images for: Multiple sclerosis risk variants regulate gene expression in innate and adaptive immune cells
Source: Life Sci Alliance. 2020 Jun 9;3(7):e202000650. doi: 10.26508/lsa.202000650 (PMC7283543; doi:10.26508/lsa.202000650)

ratio of coefficients of MS risk SNP (adjusted/unadjusted)

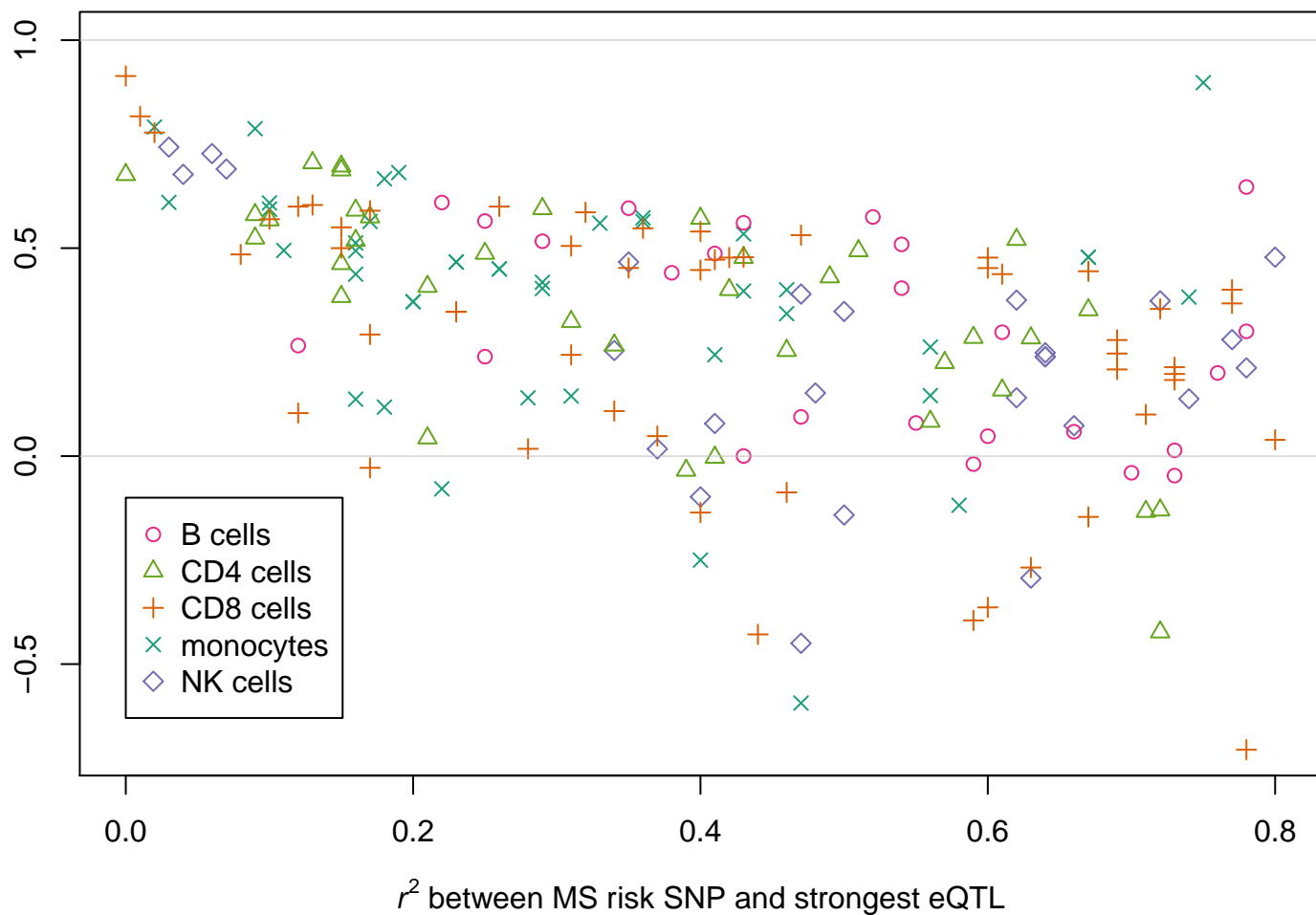

Supplement: Supplementary file 3 [file LSA-2020-00650_Supplemental_Data_3.pdf]
