# Supplementary material for: Multiple sclerosis risk variants regulate gene expression in innate and adaptive immune cells
Source: Life Sci Alliance. 2020 Jun 9;3(7):e202000650. doi: 10.26508/lsa.202000650 (PMC7283543; doi:10.26508/lsa.202000650)

Relative Log Expression (RLE) plot of monocyte samples before cross-normalization. Samples are sorted in order of processing.

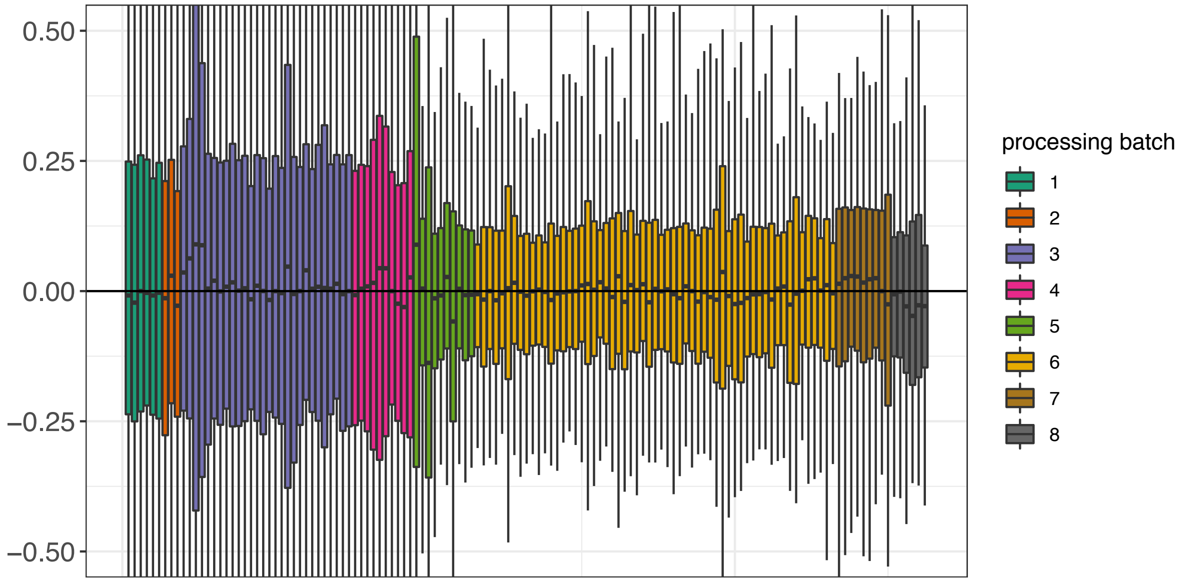


RLE plot of monocyte samples after cross-normalization using RUV-rinv with phenotype as the only factor of interest in the design matrix *X*. The leave-one-out method of cross normalization does not deal well with an individual outlier sample that has its own private pattern of unwanted variation. However RUV tests of association between genotype, phenotype and expression do not use this leave-one-out method, and hence handle individual outlier samples more effectively.

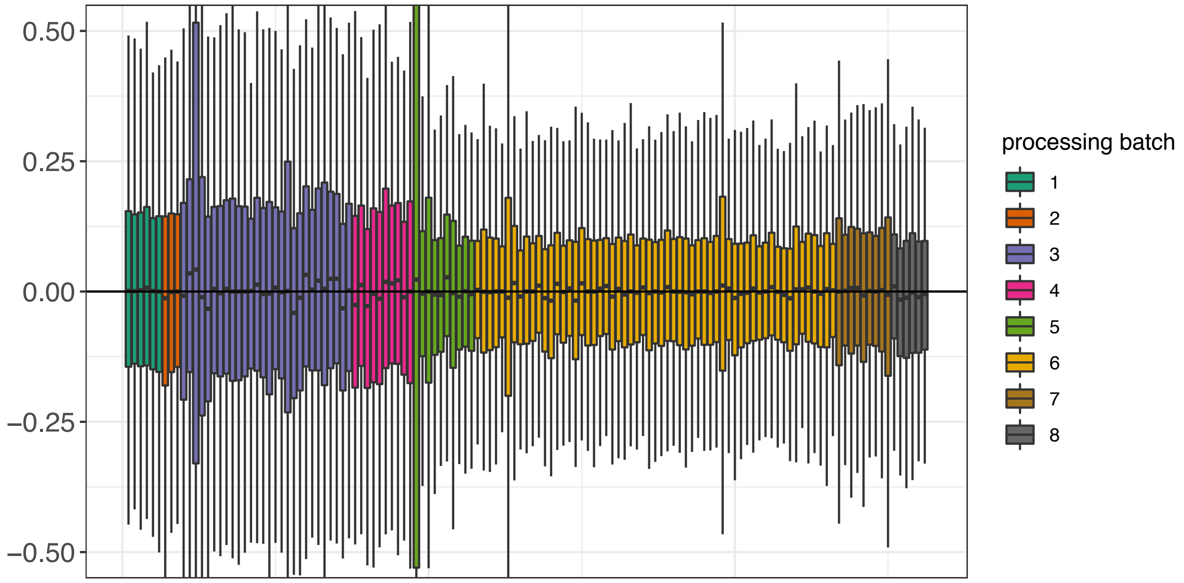

Singular value decomposition (principal components) plot of monocyte samples before cross-normalization.

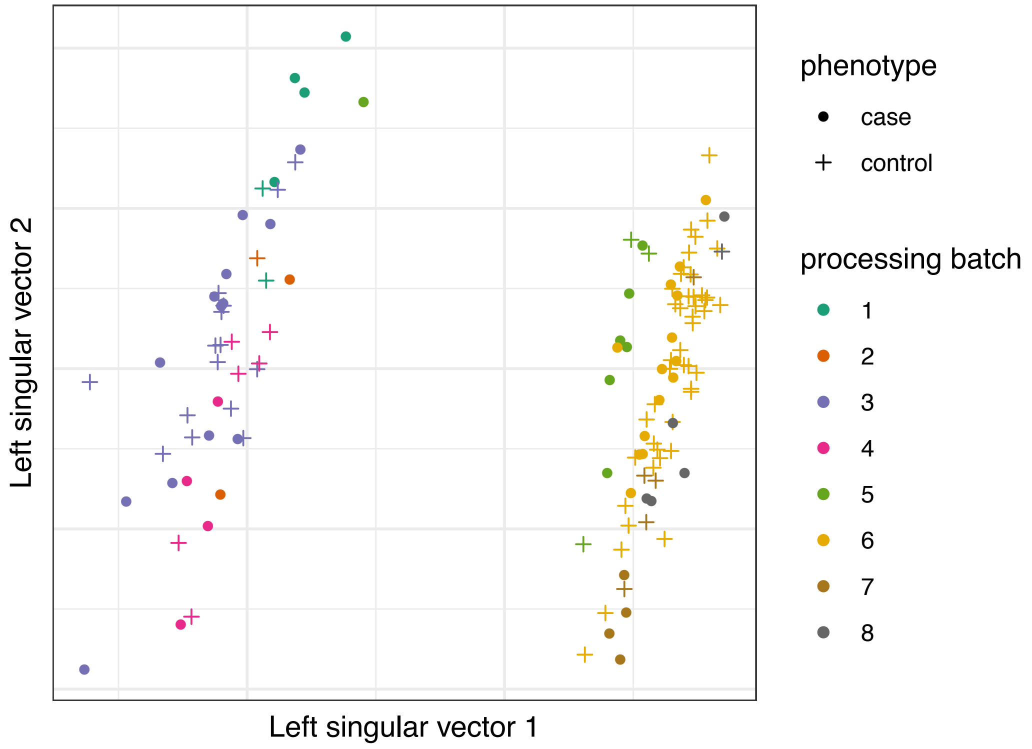


Singular value decomposition plot of monocyte samples after cross-normalization. Two outliers are not shown (one from batch 3 and one from batch 5).

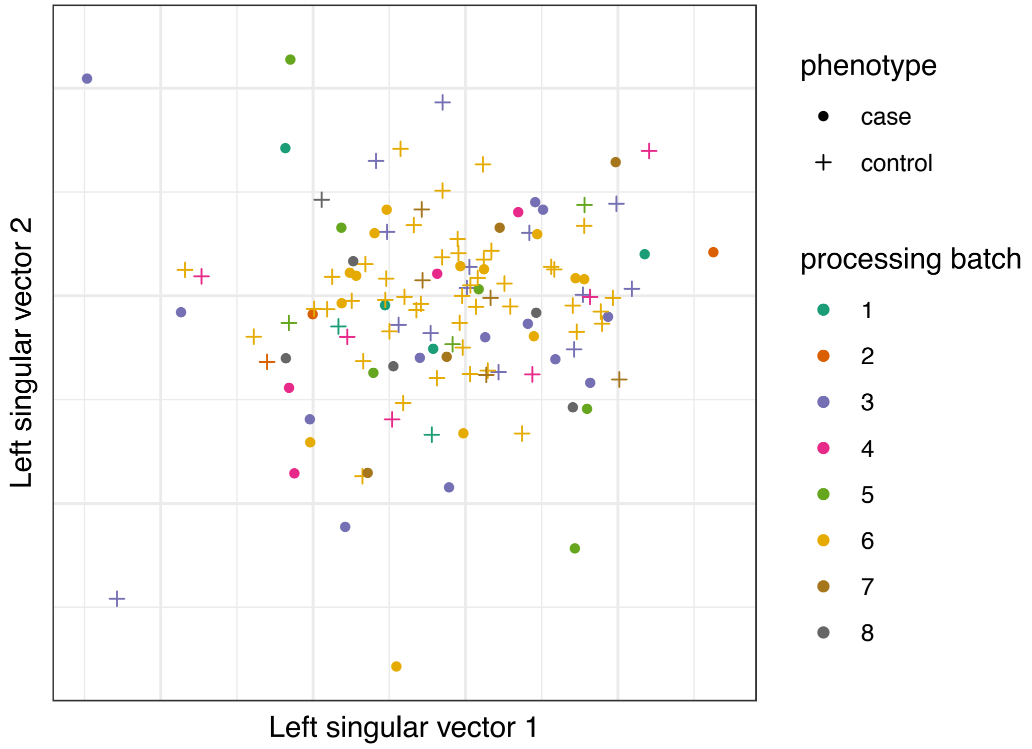

RLE plot of NK cell samples before cross-normalization.

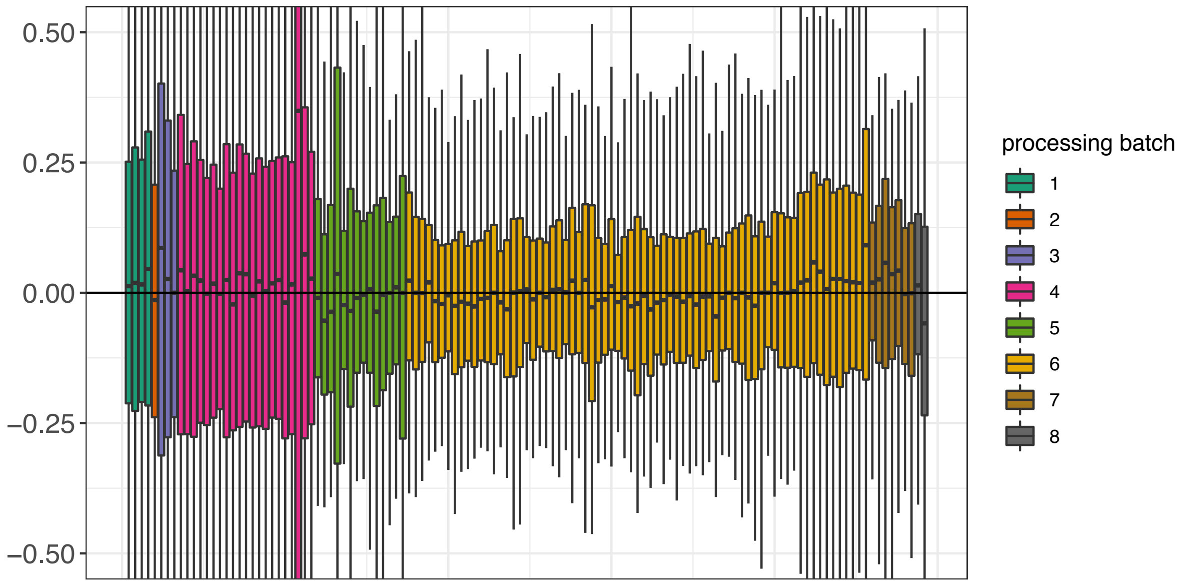


RLE plot of NK cell samples after cross-normalization.

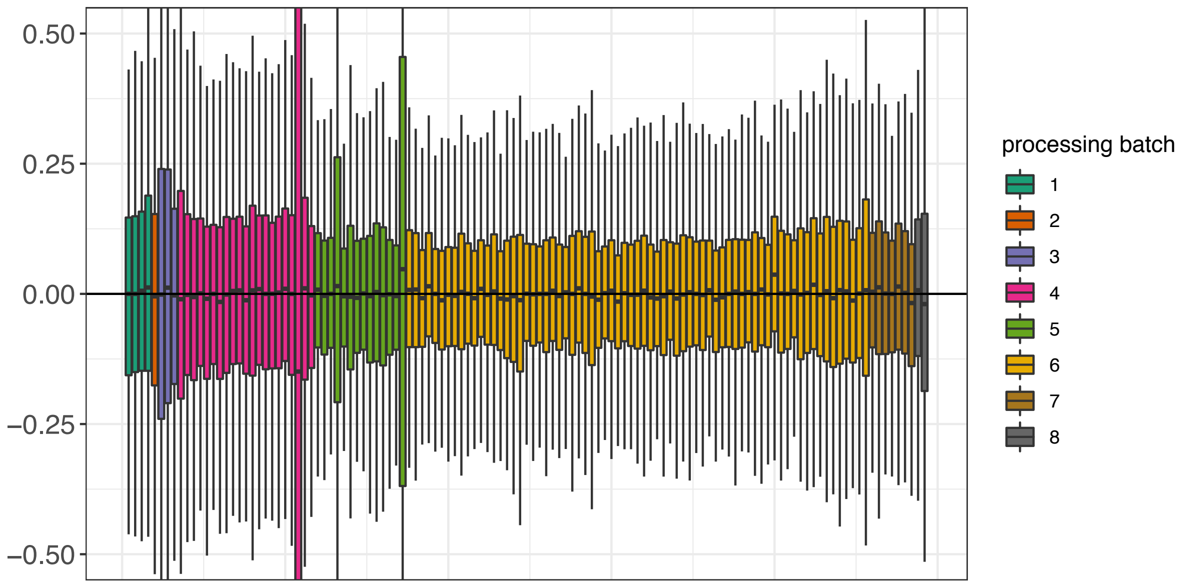

Singular value decomposition plot of NK cell samples before cross-normalization. One outlier from batch 4 is not shown.

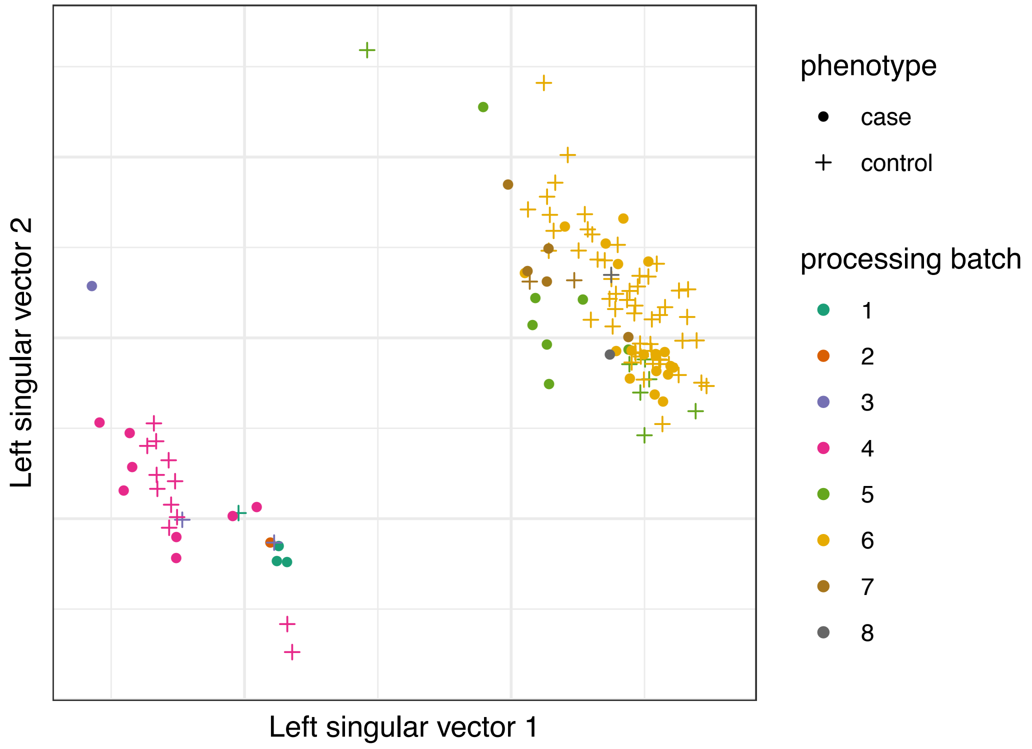


Singular value decomposition plot of NK cell samples after cross-normalization. Two outliers are not shown (one from batch 4 and one from batch 5).

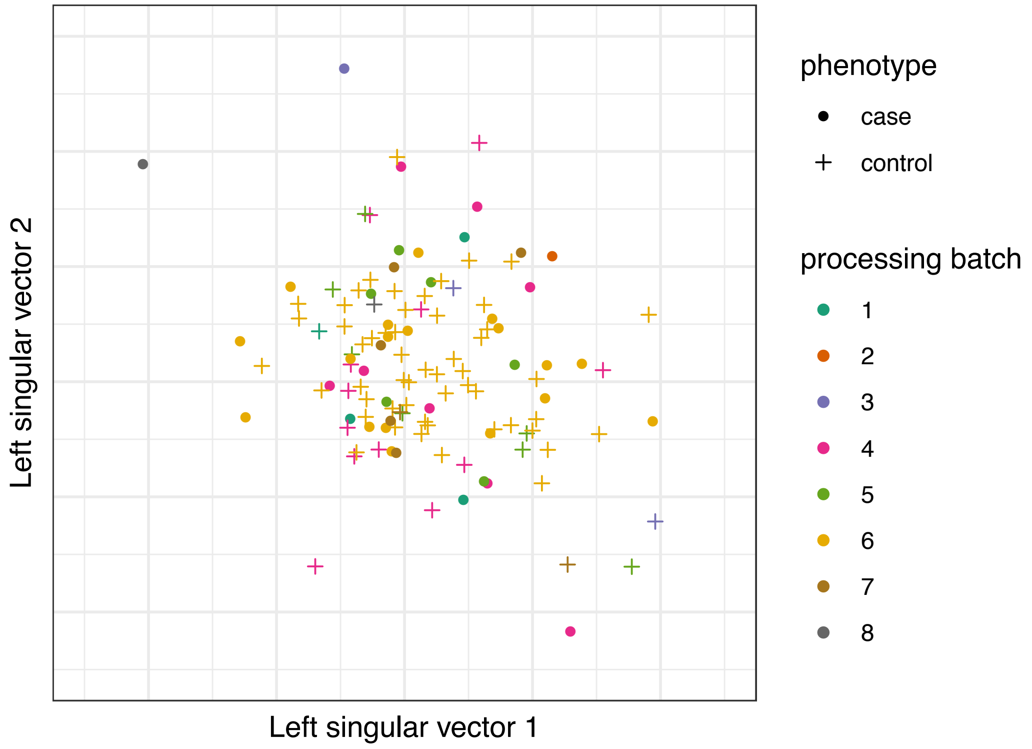

RLE plot of B cell samples before cross-normalization.

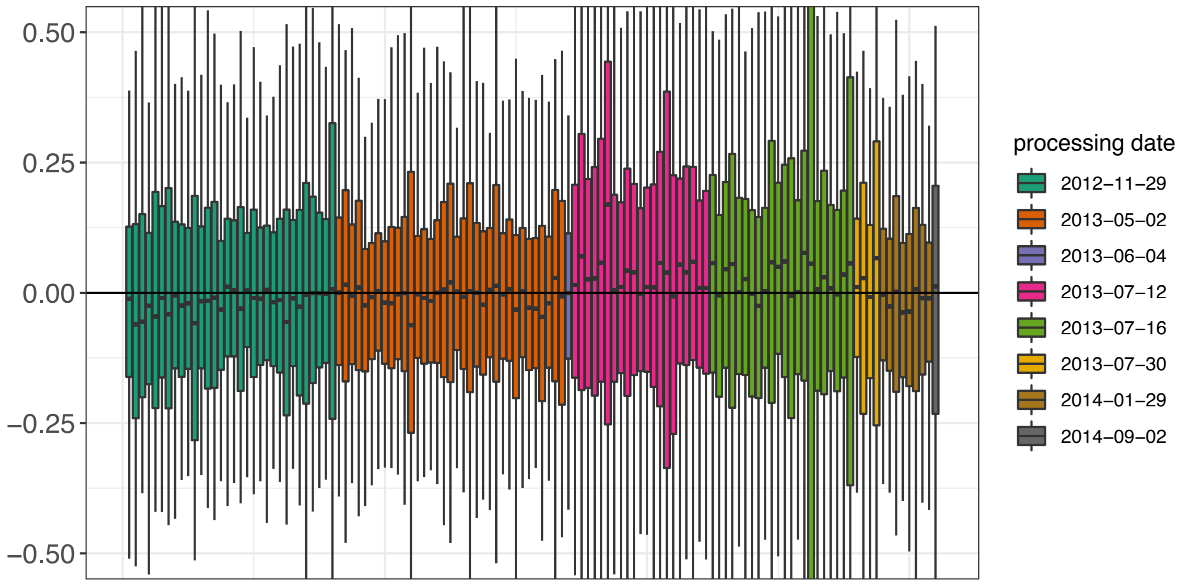


RLE plot of B cell samples after cross-normalization.

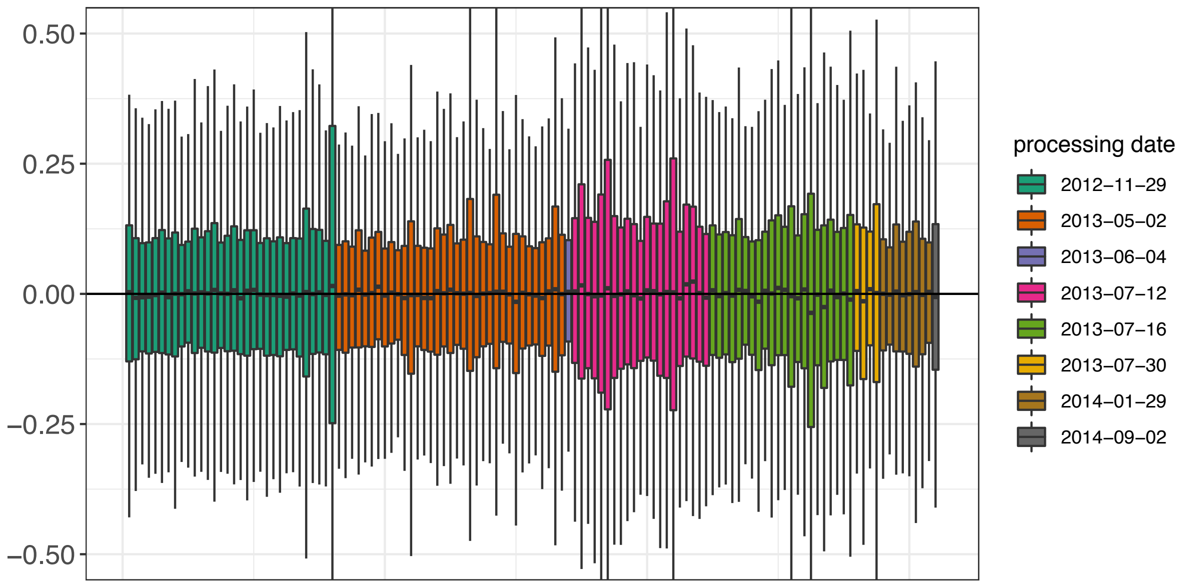

Singular value decomposition plot of B cell samples before cross-normalization.

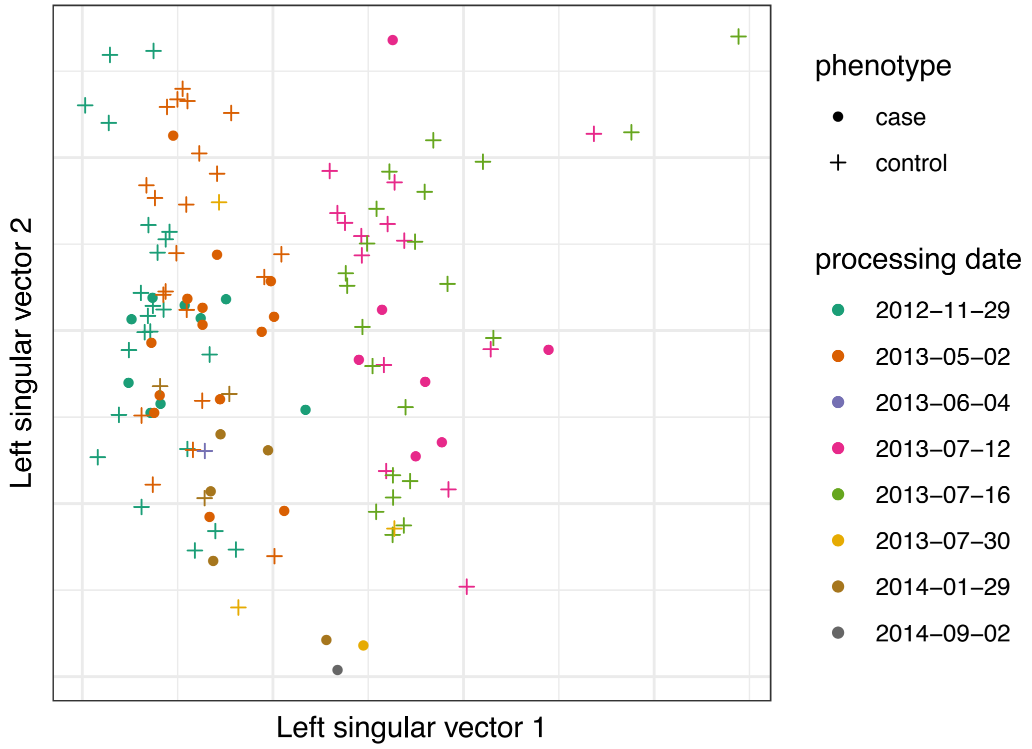


Singular value decomposition plot of B cell samples after cross-normalization. Four outliers are not shown (1 processed on 29 November 2012 and 3 processed on 12 July 2013).

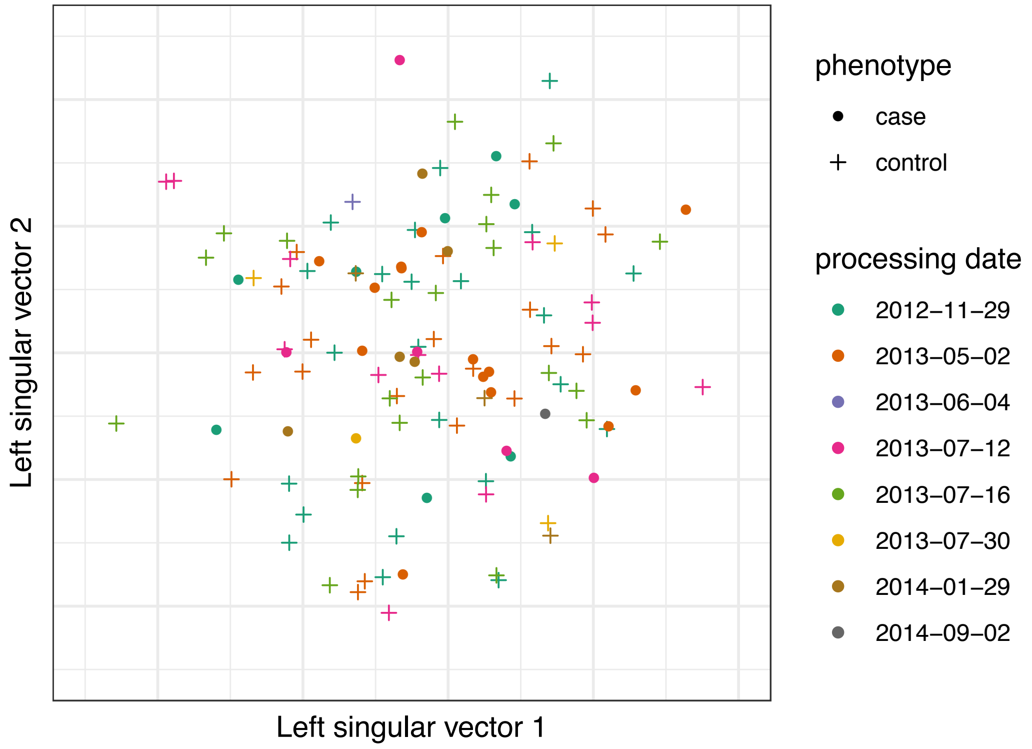

RLE plot of CD4+ T cell samples before cross-normalization.

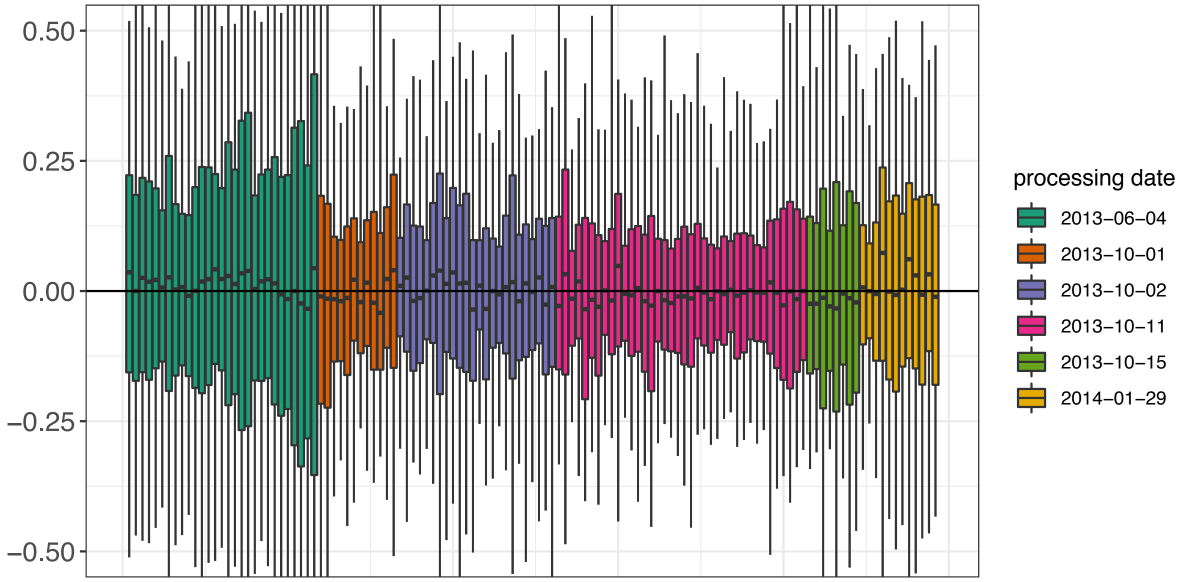


RLE plot of CD4+ T cell samples after cross-normalization.

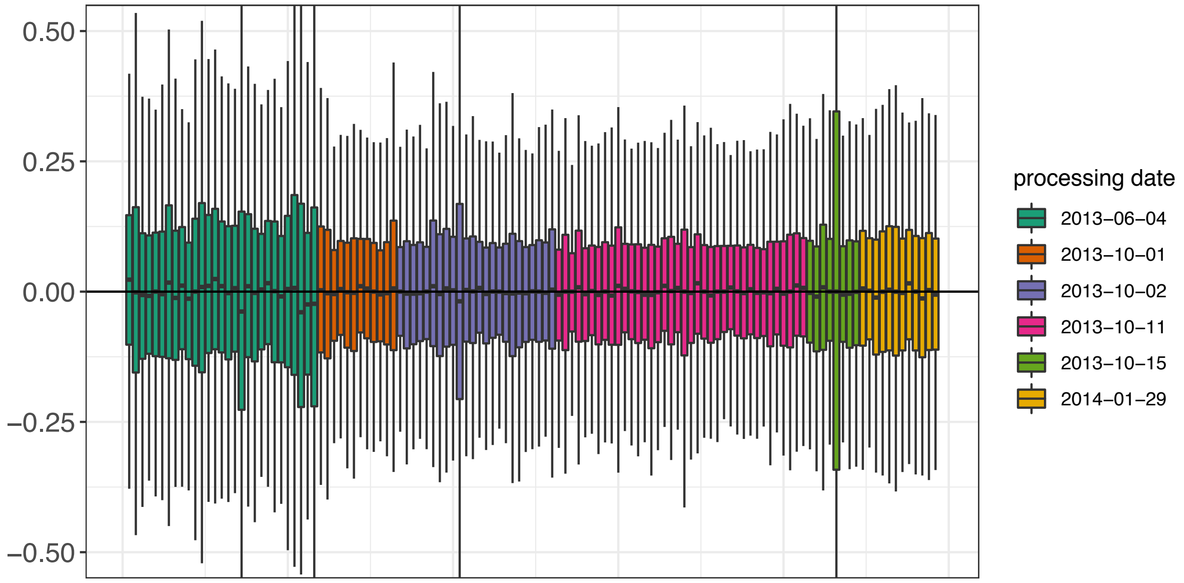

Singular value decomposition plot of CD4+ T cell samples before cross-normalization.

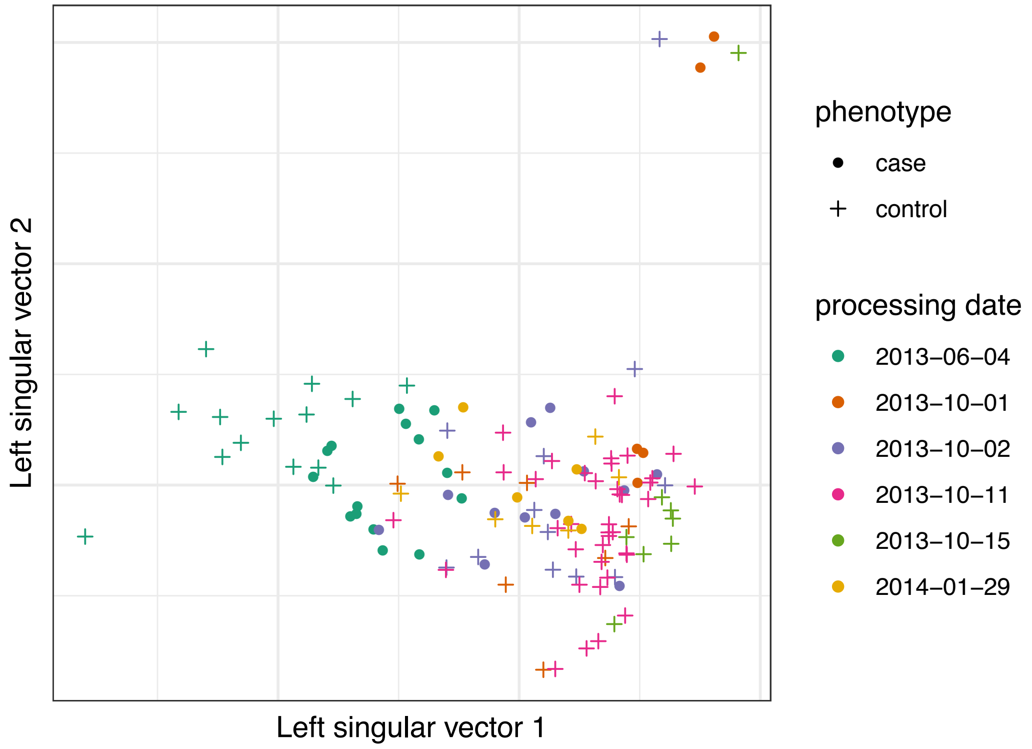


Singular value decomposition plot of CD4+ T cell samples after cross-normalization. One outlier processed on 15 October 2013 is not shown.

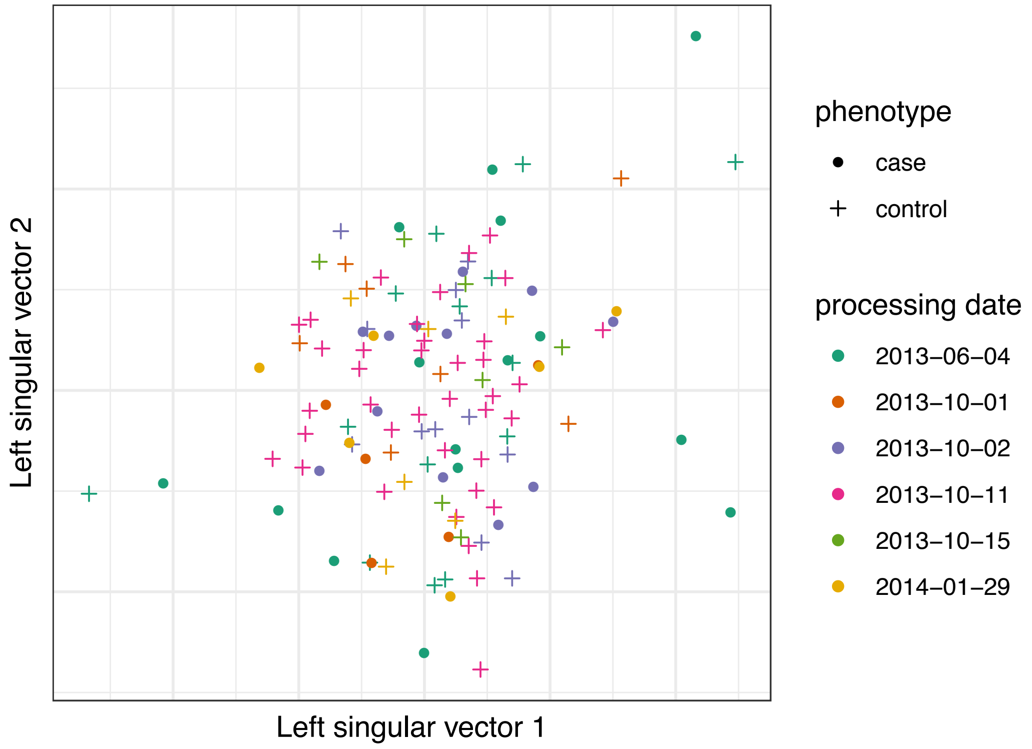

RLE plot of CD8+ T cell samples before cross-normalization.

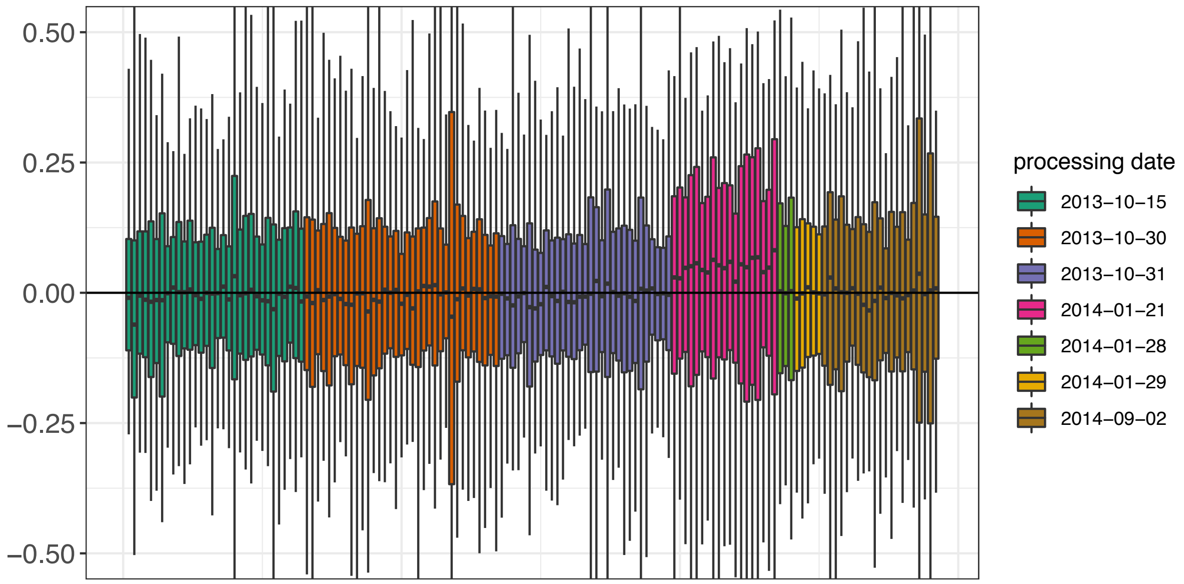


RLE plot of CD8+ T cell samples after cross-normalization.

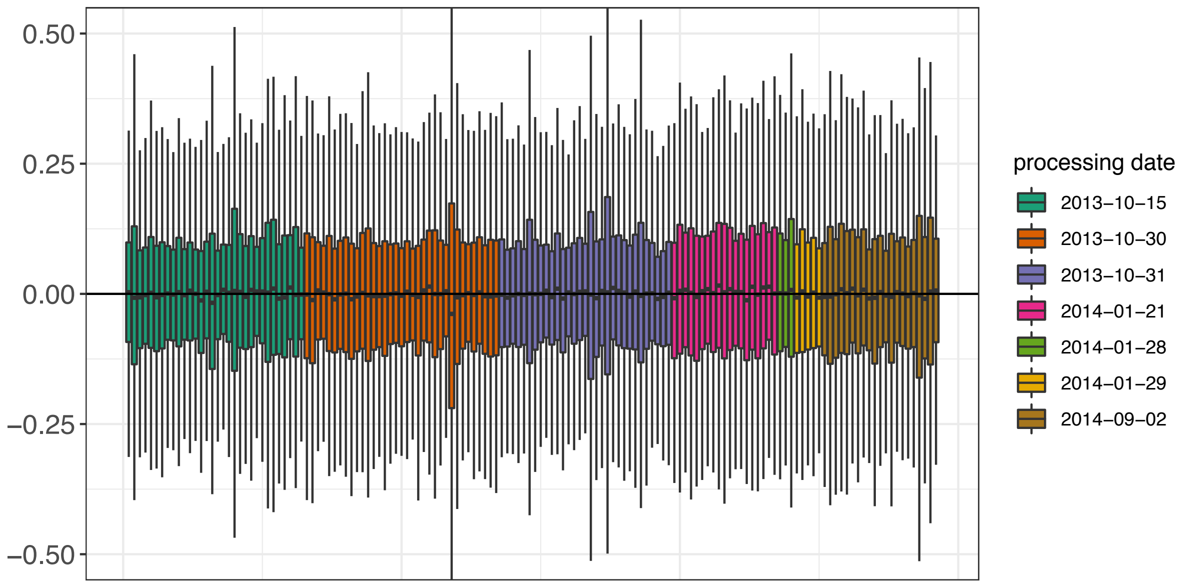

Singular value decomposition plot of CD8+ T cell samples before cross-normalization.

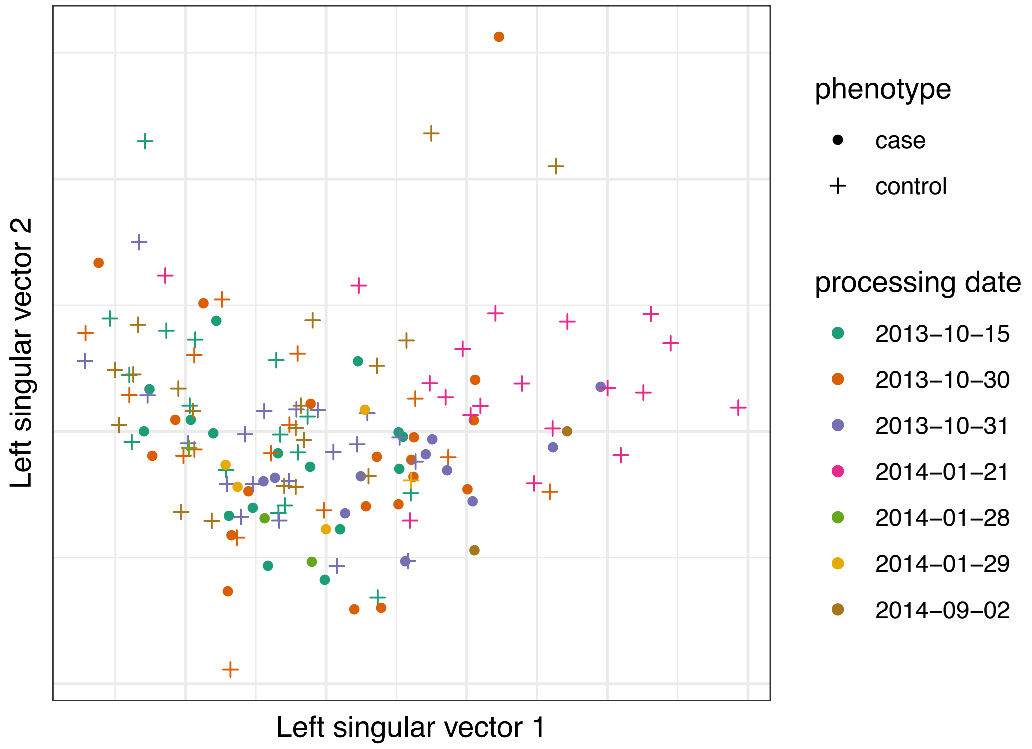


Singular value decomposition plot of CD8+ T cell samples after cross-normalization.

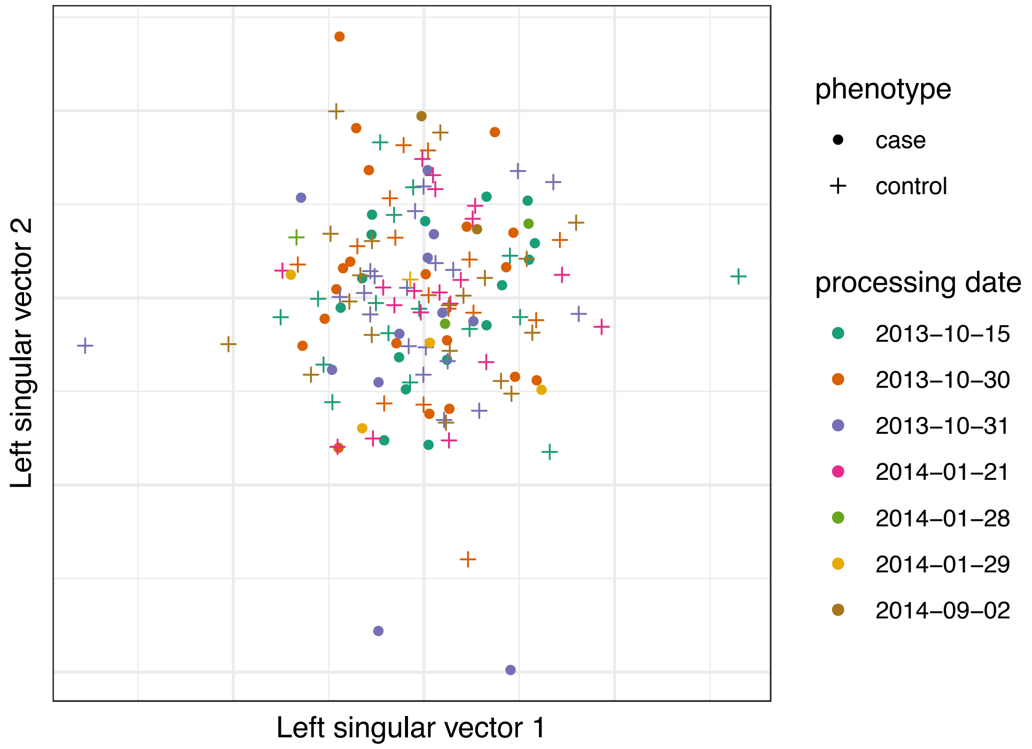

Supplement: Supplementary file 7 [file LSA-2020-00650_Supplemental_Data_7.pdf]
